# *Paraharmotrema karinganiense* n. gen., n. sp. (Digenea: Liolopidae) infecting the intestine of serrated hinged terrapin (*Pelusios sinuatus*), east African black mud turtle (*Pelusios subniger*), and South African helmeted turtle (*Pelomedusa galeata*) and a phylogenetic hypothesis for liolopid genera

**DOI:** 10.1016/j.ijppaw.2021.09.006

**Published:** 2021-10-21

**Authors:** Haley R. Dutton, Louis H. DuPreez, Misako Urabe, Stephen A. Bullard

**Affiliations:** a Aquatic Parasitology Laboratory and Southeastern Cooperative Fish Parasite and Disease Laboratory, School of Fisheries, Aquaculture, & Aquatic Sciences, College of Agriculture, Auburn University, 559 Devall Dr., Auburn, AL, 36832, USA; b African Amphibian Conservation Research Group, Unit for Environmental Sciences and Management, North-West University, Private Bag ×6001, Potchefstroom, 2520, South Africa; c South African Institute for Aquatic Biodiversity, Somerset Street, Grahamstown 6139, South Africa; d University Shiga Prefecture, Department of Ecosystem Studies, Faculty of Environmental Science, 2500 Hassaka, Hikone, Shiga, 5228533, Japan

**Keywords:** Taxonomy, Systematics, Pleurodira, Pelomedusidae, Parasite

## Abstract

We herein describe *Paraharmotrema karinganiense* n. gen., n. sp. Dutton & Bullard (Liolopidae Dollfus, 1934) from specimens infecting the intestine of the serrated hinged terrapin (*Pelusios sinuatus*), east African black mud turtle (*Pelusios subniger*) (both Nwanedzi River, Mozambique), and South African helmeted terrapin (*Pelomedusa galeata*) (North-western Zululand, KwaZulu-Natal Province, South Africa). The new genus can be easily differentiated from the other accepted liolopid genera (*Liolope* Cohn, 1902; *Helicotrema* Odhner, 1912; *Harmotrema* Nicoll, 1914; *Dracovermis* Brooks & Overstreet, 1978) by the combination of having a linguliform body approximately 6–9 × longer than wide, tegumental spines/scales, a minute ventral sucker located in the anterior 1/7−1/8 of the body, deeply lobed testes that are transverse and abut the caeca (spanning the intercaecal space), a uterus that is lateral to the anterior testis (not ventral to the anterior testis), a lobed ovary that is dextral and nearest the posterior testis, and a vitellarium that does not extend anteriad to the level of the ventral sucker and that does not fill the intercaecal space. Nucleotide sequences of large subunit ribosomal DNA (*28S*) and internal transcribed space region (*ITS2*) from all analyzed specimens of the new species were identical, respectively; the *28S* sequences differed from that of *Liolope copulans* Cohn, 1902 and from that of *Harmotrema laticaudae* Yamaguti, 1933 by 103 (8%) and 105 (8%) nucleotides, respectively. The *28S* phylogenetic analysis recovered the new genus sister to a clade comprising *L. copulans* and *H. laticaudae*. A key to liolopid genera is provided herein. The present study comprises the first nucleotide-based phylogenetic placement of *Harmotrema* and first record of a liolopid from South Africa or Mozambique. It is the first proposal of a new liolopid genus in 43 yrs, and it documents a second liolopid genus from *P. subniger* while tripling the number of liolopid turtle hosts reported from the continent of Africa.

## Highlights

• Description of *Paraharmotrema karinganiense* n. gen., n. sp. Dutton & Bullard (Liolopidae Dollfus, 1934) from African turtles.
• First nucleotide-based phylogenetic placement of *Harmotrema* and first record of a liolopid from South Africa or Mozambique.
• Documents a second liolopid genus from *P. subniger*.
• Triples the number of liolopid turtle hosts reported from the continent of Africa.
• Comments on Brooks and Overstreet 1978.

## Graphical abstract

**Figure:**
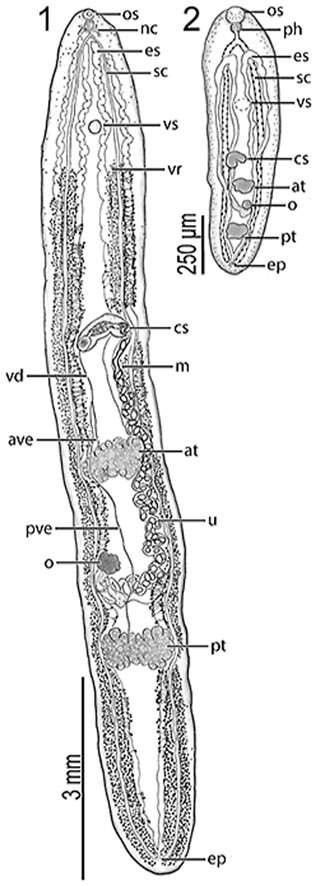

## 1 Introduction

Species of the seldom reported Liolopidae Dollfus, 1934 comprise 13 nominal species assigned to four genera (two species of *Liolope* Cohn, 1902; three of *Helicotrema* Odhner, 1912; four of *Harmotrema* Nicoll, 1914; four of *Dracovermis* Brooks and Overstreet, 1978; see also Niewiadomska, 2002) that collectively mature in the lumen of the stomach and intestine of ectothermic tetrapods (Table 1; Table 2). Infections have been reported from all continents but Europe, Australia, and Antarctica. During a recent parasitological expedition led by LdP and including HRD and SAB in South Africa and Mozambique, we collected specimens of a rather large trematode from the intestinal lumen of 3 turtles (*Pelusios sinuatus* (Smith, 1838)*, Pelusios subniger* (Bonnaterre, 1789), *Pelomedusa galeata* (Schoepff, 1792)). We herein describe these trematode specimens as a new species, propose a new genus for this new species, and present a phylogenetic hypothesis for Liolopidae based on sequences of the large subunit ribosomal DNA (*28S*).

**Table 1:** Records for Liolopidae (type species in bold; cercarial infections*).

Liolopid species	Host	Locality	Reference
***Liolope copulans*****Cohn (1902)**	*Andrias japonicus* (Temminck, 1836) (Urodela: Cryptobranchidae), Japanese giant salamander	imported from Japan to Europe	Cohn (1902)
		Yoshii River, Okayama Prefecture, Honshu, Japan	Kagawa and Kuyama (1935)
		Hiroshima Prefecture, Honshu, Japan	Ozaki & Okuda (1951)
		Mt. Kasaoka in Okayama Prefecture, Japan	Brooks & Overstreet (1978)
		Suma Aqualife Park, Kobe, Hyogo Prefecture, Honshu Island, Japan	Hasegawa et al. (2002)
		Yoshii River, Okayama, Japan	Baba et al. (2011)
		Akame, Nabari City, Mie Prefecture, Honshu, Japan	Baba et al. (2011)
		Okuyama River, Kobe City, Hyogo Prefecture, Japan	Baba et al. (2011)
		Hatsuka River, Hyogo Prefecture, Japan	Baba et al. (2011)
		Asa Zoological Park, Hiroshima City, Hiroshima Prefecture (originated from Ota River, Hiroshima Prefecture), Japan	Tanaka et al. (2016)
	*Andrias davidianus* (Blanchard, 1871) (Urodela: Cryptobranchidae), Chinese giant salamander	Guiyang Market in Kweichow, Guizhou Province	Chin et al. (1974)
		Kyoto City, Kyoto Prefecture, Japan (introduced)	Tsuchida et al. (2021)
	*Andrias davidianus × A. japonicus,* a hybrid of the Chinese giant salamander and the Japanese giant salamander	Kyoto City, Kyoto Prefecture, Japan	Tsuchida et al. (2021)
	*Semisulcospira libertina (Gould, 1859) (Gastropoda: Semisulcospiridae)	Nabari River, Mie and Nara Prefectures, Japan	Makita et al. (1996) (in Baba et al., 2011)
*L. dollfusi*Skrjabin (1962)	*Pelusios subniger* (Bonnaterre, 1789) (Pleurodira: Pelomedusidae), East African black mud turtle	Gabon, dissection at the Paris Museum	Dollfus (1950) (*in*Skrjabin 1962)
***Helicotrema magniovatum*****Odhner (1912)**	*Iguana iguana* (Linnaeus 1758) (Iguania: Iguanidae)*,* common green iguana	South America	Odhner (1912)
*H. spirale* (Diesing, 1850) Odhner (1912) (originally *Monostomum*)	*Iguana iguana* (Linnaeus 1758) (Iguania: Iguanidae)*,* common green iguana	Caieiras, State of São Paulo, Brazil	Diesing (1850)
	*Peltocephalus dumerilianus* (Schweigger, 1812) (Pleurodira: Podocnemididae), big-headed side neck turtle	Orinoco River, Marabitanas, Brazil	Diesing (1850)
	*Chelonoidis denticulatus* (Linnaeus, 1766) (Cryptodira: Testudinidae), yellow-footed tortoise	Borba, Brazil	Diesing (1850)
		Iquitos, Peru	Julca et al. (2014)
*H. asymmetricum*Travassos (1922)	*Iguana iguana* (Linnaeus 1758) (Iguania: Iguanidae)*,* common green Iguana	Mato Grosso, Brazil	Travassos (1922)
	*Rhinoclemmys melanosterna* (Gray, 1861) (Cryptodira: Geoemydidae), Colombian wood turtle	Panama	Travassos (1922)
*Helicotrema* sp.	*Iguana iguana* (Linnaeus, 1758)	Cuiaba', Mato Grosso, Brazil	Avila (2011)
***Harmotrema infecundum*****Nicoll (1914)**	*Grayia smythii* (Leach, 1818) (Serpentes: Colubridae), Smith's African water snake	London Zoological Gardens (host from Africa)	Nicoll (1914)
*H. laticaudae*Yamaguti (1933)	*Laticauda laticaudata* (Linnaeus 1758) (Serpentes: Elapidae), blue-lipped sea krait	Isigaki Zima, Okinawa, Japan	Yamaguti (1933)
	*Laticauda semifasciata* (Reinwardt, 1837) (Serpentes: Elapidae), black-banded sea krait	Amami Island, Kagoshima, Japan	Telford (1967)
		Jeju-do, South Korea	Choe et al. (2020)
		Ishigaki Island (=Isigaki Zima), Okinawa, Japan	Present study
	*Hydrophis major* (Shaw, 1802) (Serpentes: Elapidae), olive-headed sea krait	Queensland, Australia	Brooks & Overstreet (1978)
	*Aipysurus laevis* (Lacépède, 1804) (Serpentes: Elapidae), olive sea krait	Queensland, Australia	Brooks & Overstreet (1978)
	"black and white-ringed sea snake"	Queensland, Australia	Brooks & Overstreet (1978)
*H. eugari*Tubangui and Masilungan (1936)	*Naja philippinensis* (Taylor, 1922) (Serpentes: Elapidae), Philippine cobra	Binan, Laguna, Luzon, Phillippines	Tubangui and Masilungan (1936); Fischthal and Kuntz (1967)
	*Cerberus rynchops* (Schneider, 1799) (Serpentes: Homalopsidae), New Guinea bockadam	Zamboanga, Mindanao Island, Philippines	Fischthal & Kuntz (1967)
*H. indica*Chattopadhyaya (1970)	*Hydrophis schistosa* Daudin, 1803 (Serpentes: Elapidae), Beaked sea snake	Bombay, India	Chattopadhyaya (1970)
***Dracovermis occidentalis*****Brooks and Overstreet (1978)**	*Alligator mississippiensis* (Daudin, 1801) (Crocodilia: Alligatoridae), American alligator	Cameron Parish, LA, USA	Brooks & Overstreet (1978)
		Southeast TX, USA	Scott et al. (1997), 1999
		Horn Island, MS, USA	Brooks & Overstreet (1978)
*D. brayi*Brooks and Overstreet (1978)	*Mecistops cataphractus* (Cuvier, 1825) (Crocodilia: Crocodylidae), West African slender-snouted crocodile	Belgian Congo, Africa	Baylis (1940) (in Brooks and Overstreet, 1978)
*D. rudolphii* (Tubangui and Masilungan, 1936) Brooks and Overstreet (1978) (originally *Harmotrema)*	*Crocodylus porosus* (Schneider, 1801) (Crocodilia: Crocodylidae), saltwater crocodile	Palawan, Phillippines	Tubangui and Masilungan (1936); Brooks and Overstreet (1978)
*D. nicollii* (Mehra, 1936) Brooks and Overstreet (1978) (originally *Harmotrema)*	*Gavialis gangeticus* (Gmelin, 1789) (Crocodilia: Gavialidae), gharial	Allahabad, India	Mehra (1936); Brooks and Overstreet (1978)
***Paraharmotrema karinganiensis* Dutton & Bullard n. gen., n. sp**.	*Pelusios sinuatus* (Smith, 1838) (Pleurodira: Pelomedusidae), serrated hinged terrapin	Nwanedzi River, Karingani Game Reserve, Maputo Province, Mozambique	present study
		Phongolo River, near Ndumo Game Reserve, KwaZulu-Natal Province, South Africa	present study
	*Pelusios subniger* (Bonnaterre, 1789) (Pleurodira: Pelomedusidae), East African black mud turtle	Temporal ponds, Karingani Game Reserve, Maputo Province, Mozambique	present study
	*Pelomedusa galeata* (Schoepff, 1792) (Pleurodira: Pelomedusidae), South African helmeted terrapin	Near Tembe Elephant Park, KwaZulu-Natal Province, South Africa	present study

**Table 2:** Key to accepted genera of Liolopidae.

1a.	Testes in anterior half of body	*Helicotrema*Odhner, 1912
1b.	Testes in posterior half of body	2
2a.	Cirrus sac abutting ventral sucker	*Liolope*Cohn, 1902
2b.	Cirrus sac not abutting ventral sucker	3
3a.	Testes near, in posterior 1/3 of body	*Dracovermis*Brooks and Overstreet, 1978
3b.	Testes far apart, not limited to posterior 1/3 of body	4
4a.	Testes smooth, not deeply lobed	*Harmotrema*Nicoll, 1914
4b.	Testes deeply lobed	*Paraharmotrema* Dutton & Bullard n. gen.

## 2 Materials and methods

### 2.1 Specimen collection and preparation

Turtles were sampled during March 2020 from Karingani Game Reserve (KGR), Maputo province, Mozambique (24°20′8.09″S 32°15′42.0″E) and a roadside borrow pit filled with water in north-western Zululand, Kwa-ZuluNatal Province, South Africa (SA) (27°00′52.8″S 32°08′30.1″E). The digestive tract of each turtle was excised intact, sliced longitudinally to expose the lumen, immersed in saline, and examined with stereo-dissecting microscopes each equipped with a fiber optic light source. Trematodes intended for morphology were observed microscopically, heat-killed on glass slides using a butane hand lighter under no coverslip pressure, fixed in 10% neutral buffered formalin (nbf), rinsed with water, stained in Van Cleave's hematoxylin with several drops of Ehrlich's hematoxylin, dehydrated through a graded series of EtOHs, made basic at 70% EtOH, exposed to a few drops of lithium carbonate and butyl-amine to ensure specimens are basic, dehydrated in absolute EtOH and xylene, cleared with clove oil, and permanently mounted on glass slides using Canada balsam (Dutton et al., 2019). The resulting whole mounts were examined and illustrated with the aid of an Olympus BX53 with DIC (Tokyo, Japan) with drawing tube and a Ken-A-Vision X1000 Micro-projector (Raytown, MO, USA). Measurements were obtained with a calibrated ocular micrometer (as straight-lines along the course of each duct) and are reported in micrometres (μm) as the range followed by the mean, +/− standard deviation, and sample size in parentheses. Two specimens were processed for scanning electron microscopy (SEM) as per Bullard et al. (2019). Type and voucher materials of the new species were deposited in the National Museum of Natural History's Invertebrate Zoology Collection (USNM, Smithsonian Institution, Washington, D. C.). Turtle scientific and common names follow Rhodin et al. (2017), and higher classification of turtles follows Pereira et al. (2017).

A black-banded sea krait (*Laticauda semifasciata* (Reinwardt, 1837) (Serpentes: Elapidae)) was collected from Sakieda (Ishigaki Island, Okinawa Prefecture, Japan) on 8 September 2016 by Takahide Sasai, maintained alive in the Suma Aqualife Park, and necropsied there on 19 September 2016 by MU. Five adult specimens of *H. laticaudae* were collected from the intestine of that individual sea krait: one adult specimen of *H. laticaudae* was fixed in 95% EtOH and processed for DNA extraction and sequencing; 3 were stained in Heidenhain's iron hematoxylin, and 1 was retained in 90% EtOH.

### 2.2 Phylogenetic analysis

Total genomic DNA (gDNA) was extracted from 3 EtOH-preserved and microscopically identified specimens of the new species (2 adults of the new species from *P. galeata* in SA; 1 juvenile specimen of the new species from *P. subniger* in KGR) and from the EtOH-preserved and microscopically identified specimen of *H. laticaudae* from *L. semifasciata* using DNeasy™ Blood and Tissue Kit (Qiagen, Valencia, California) as per the manufacturer's protocol except that the proteinase-K incubation period was extended overnight, and 100 μL of elution buffer was used to increase the final DNA concentration. The nuclear large subunit ribosomal DNA (*28S*) and the internal transcribed spacer-2 region (*ITS2*) were amplified using the primer set of Orélis-Ribeiro et al. (2017). PCR amplifications were performed according to Dutton et al. (2019). DNA sequencing was performed by Genewiz, Incorporated (South Plainfield, New Jersey, USA). Sequence assembly and analysis of chromatograms were performed with Geneious version 2019.2.3 (http://www.geneious.com). All nucleotide sequence data were deposited in GenBank (OL413003−OL413009). The *28S* phylogenetic analysis included 3 identical sequences from the 2 hosts (see above) plus the single available liolopid in GenBank (Baba et al., 2011). Other taxa included in the analysis were informed by Baba et al. (2011) and Hernández-Mena et al. (2017). Sequences were aligned with the multiple alignment tool using fast Fourier transform (MAFFT) (Katoh and Standley, 2013) and trimmed to the length of the shortest sequence (1223 [*28S*] base pairs). JModelTest 2 version 2.1.10 was implemented to perform statistical selection of the best-fit models of nucleotide substitution based on Bayesian Information Criterion (BIC) (Darriba et al., 2012). Aligned sequences were reformatted (from .fasta to .nexus) using the web application ALTER (Glez-Peña et al., 2010) to run Bayesian inference (BI). BI was performed in MrBayes version 3.2.5 (Ronquist and Huelsenbeck, 2003) using substitution model averaging (“nst-mixed”) and a gamma distribution to model rate-heterogeneity. Defaults were used in all other parameters. Three independent runs with 4 Metropolis-coupled chains were run for 5,000,000 generations, sampling the posterior distribution every 1000 generations. Convergence was checked using Tracer v1.6.1 (Rambaut et al., 2014) and the “sump” command in MrBayes: all runs appeared to reach convergence after discarding the first 25% of generation as burn-in. A majority rule consensus tree of the post burn-in posterior distribution was generated with the “sumt” command in MrBayes. The inferred phylogenetic tree was visualized using FigTree v1.4.4 (Rambaut et al., 2014) and further edited for visualization purposes with Adobe Illustrator (Adobe Systems).

## 3 Results

Family Liolopidae Dollfus, 1934.

### *3.1* *Paraharmotrema* Dutton & Bullard

#### 3.1.1 Generic diagnosis

Body of adult extremely elongate, linguliform, approximately 6–9 × longer than wide, having tegumental spines/scales. Oral sucker slightly smaller than ventral sucker, ventral, subterminal, lacking unique spines/scales. Ventral sucker minute, weakly muscular, in anterior 1/7−1/8 of body, inter-caecal, not spanning inter-caecal space. Intestine comprising paired caeca extending sinuously posteriad approximately in parallel with lateral body margin and nearly reaching posterior body extremity, lacking diverticula and lateral out-pocketings, bowing laterad at level of testes. Gonads inter-caecal, in posterior half of body, posterior to male and female terminal genitalia. Testes deeply lobed, transverse (markedly wider than long), tandem, medial, closely flanked by caeca, delimiting male and female proximal genitalia, in posterior half of body. Cirrus sac massive (spanning breadth of inter-caecal space), transverse or oblique, between ventral sucker and anterior testis, containing bipartite seminal vesicle, pars prostatica, and spined eversible cirrus. Common genital pore sinistral, ventral to sinistral caeca, post-acetabular, pre-testicular. Ovary lobed, dextral, inter-testicular, inter-caecal, nearest posterior testis. Vitellarium co-distributing with caeca from posterior to level of ventral sucker posteriad to posterior body end, not extending anteriad to level of intestinal bifurcation, not extending mediad far beyond excretory ducts. Uterus extremely elongate, sinuous, lateral to anterior testis (not ventral to anterior testis), containing many operculate eggs. Excretory system comprising lasso configuration dextrally and sinistrally (excretory system having a pair of ducts, each having an anterior cyclocoel-like portion and a posteriorly-directed collecting duct); excretory pore dorsal (subterminal). Intestinal parasites of turtles.

#### 3.1.2 Differential diagnosis

Body of adult extremely elongate, linguliform (vs. ovoid or pyriform in *Liolope* and *Dracovermis*), approximately 6–9 × longer than wide, having tegumental spines/scales (vs. spines/scales absent in *Helicotrema* and *Dracovermis*; present in some species of *Liolope* and *Harmotrema*). Ventral sucker minute, weakly muscular (vs. strongly muscular in *Liolope* and *Dracovermis*), in anterior 1/7−1/8 of body (vs. anterior 1/3 in *Liolope*; anterior 1/20 in *Helicotrema*, anterior 1/3−1/7 in *Harmotrema*, and middle 1/3 in *Dracovermis*), inter-caecal, not spanning inter-caecal space (vs. spanning intercaecal space in some species of *Liolope* and *Dracovermis*). Intestine bowing laterad at level of testes (vs. not bowing laterad in *Liolope, Helicotrema*, *Harmotrema* and *Dracovermis*). Testes deeply lobed (vs. smooth in *Liolope* [except *L. dollfusi* that has lobed testes]*, Helicotrema*, *Harmotrema* and *Dracovermis*), transverse (markedly wider than long) (vs. spheroid in *Liolope, Helicotrema* and *Harmotrema*), in posterior half of body (vs in anterior half of body in *Helicotrema*). Ovary lobed, dextral, nearest posterior testis (vs. nearest anterior testis in *Liolope* or abutting testes in *Dracovermis*). Vitellarium distributing from posterior to level of ventral sucker to posterior body end, not extending mediad far beyond excretory ducts (vs. vitellarium extending anteriad to ventral sucker in *Liolope*, *Harmotrema infecundum* (type species), and *Dracovermis*). Uterus extremely elongate, lateral to anterior testis (not ventral to anterior testis), containing many operculate eggs (vs. uterus short in *Liolope* and *Dracovermis*). Excretory pore dorsal (subterminal) (vs. terminal in *Liolope*). Intestinal parasites of turtles.

#### 3.1.3 Taxonomic summary

*Type-species: Paraharmotrema karinganiense* n. sp.

*Etymology*: The genus name refers to the similarity between the new genus and *Harmotrema*.

### 3.2 Paraharmotrema karinganiense n. sp. Dutton & Bullard

#### 3.2.1 Diagnosis of adult specimens (based on six whole-mounted specimens and one hologenophore; USNM coll. nos. 1659278c\, 1659286)

**Figs. 1–2:**
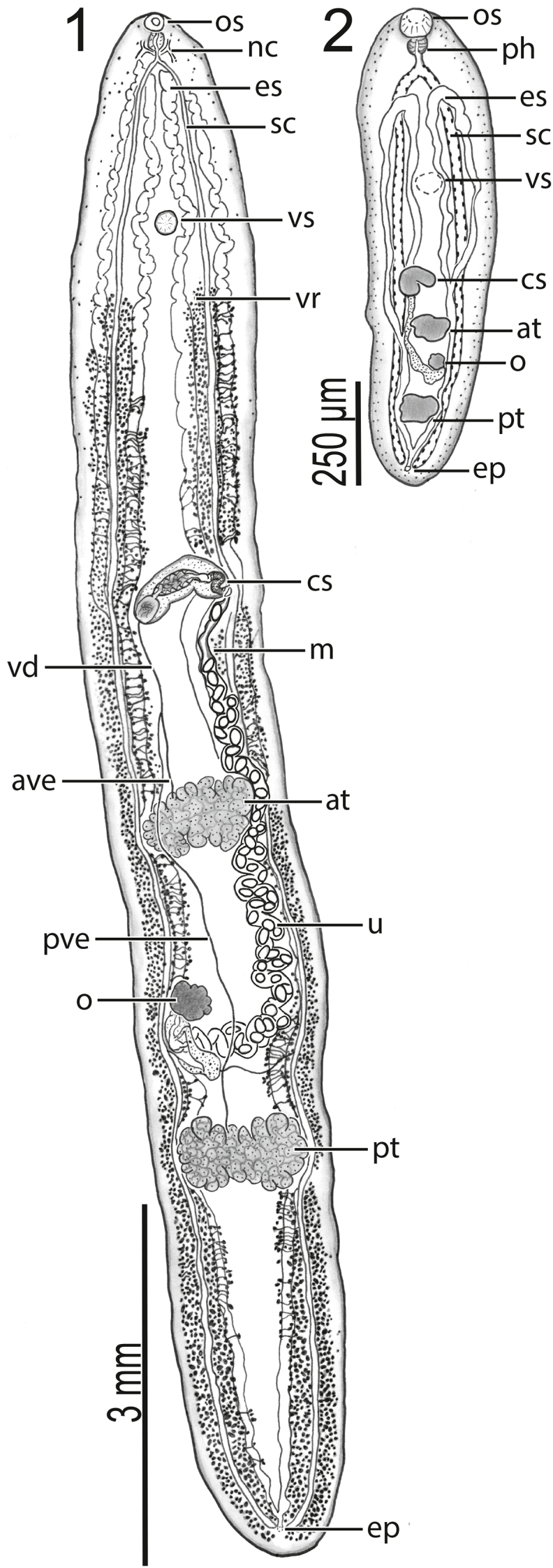
*Paraharmotrema karinganiense* Dutton & Bullard n. sp. (Digenea: Liolopidae). (**1**) Body of adult (holotype, USNM No. 1659278) from intestine of serrated hinged terrapin, *Pelusios sinuatus* (Smith 1838) (Pleurodira: Pelomedusidae), ventral view. (**2**) Body of juvenile (paratype, USNM No. 1659285) from intestine of east African black mud turtle, *Pelusios subniger* (Bonnaterre, 1789) (Pleurodira: Pelomedusidae), dorsal view. Oral sucker (os), pharynx (ph), nerve commissure (nc), excretory system (es), sinistral caecum (sc), ventral sucker (vs), vitellarium (vr), cirrus sac (cs), metraterm (m), vas deferens (vd), anterior vas efferens (ave), anterior testis (at), uterus (u), posterior vas efferens (pve), ovary (o), posterior testis (pt), and excretory pore (ep).

Body 8950−12,675 (10,583 ± 1590; 6) long, 1375−1625 (1504 ± 106; 7) in maximum width at level of ovary (Fig. 1−2), 5.6–8.5 × (7.1 ± 1.2; 6) longer than wide. Body with ventral concavity in specimens with edges curved ventrally (Fig. 1). Tegumental scales distributing around anterior end and oral sucker only (Fig. 7−9). Oral sucker 130–190 (157 ± 20; 6) in diameter or 1–2% (1% ± 0%; 6) of body length or 76–94% (87% ± 9%; 6) of ventral sucker diameter, 160–180 (168 ± 10; 6) wide or 11–13% (11% ± 1%; 6) of maximum body width (Fig. 1−2). Ventral sucker 160–250 (182 ± 34; 6) long or 1–2% (2% ± 0%; 6) of body length, 190–230 (208 ± 16; 6) wide or 28–31% (29% ± 1%; 6) of maximum body width, positioning 1250−1675 (1417 ± 158; 6) or 12–15% (13% ± 1%; 6) of body length from anterior end of body (Fig. 5−6). Nerve commissure 260–330 (286 ± 27; 6) or 2–3% (3% ± 0%; 6) of body length from anterior body end (Fig. 1). Pharynx 120–140 (127 ± 8; 6) or 68–80% (71% ± 5%; 6) of oesophagus length, 105–135 (118 ± 11; 6) wide or 2–12 × (6 ± 3; 6) wider than maximum oesophagus width (Fig. 1−2). Oesophagus 150–200 (179 ± 17; 6) long, 10–45 (23 ± 12; 6) wide; surrounded by glandular cells. Intestine bifurcating 300–380 (326 ± 30; 6) or 3% (3% ± 0%; 6) of body length from anterior body end (Fig. 1−2).

**Figs. 3–4:**
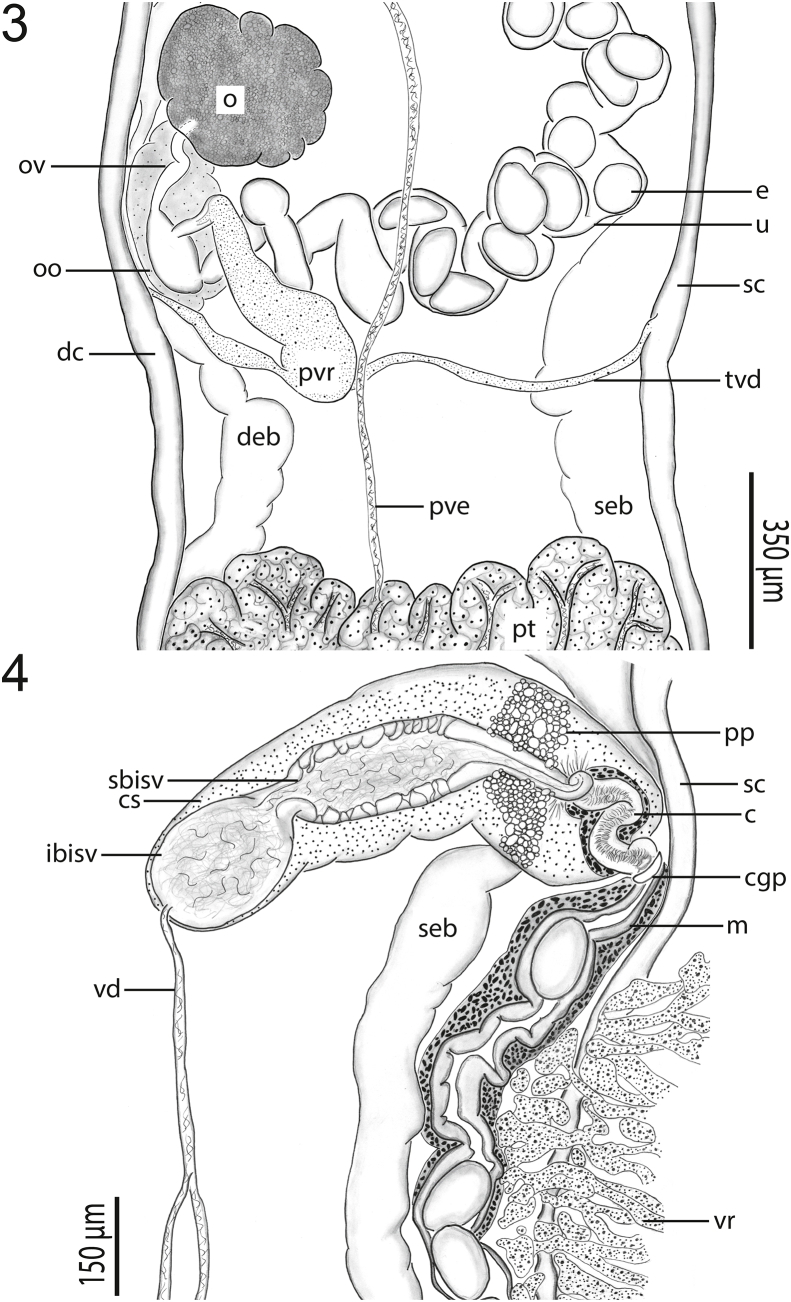
*Paraharmotrema karinganiense* Dutton & Bullard n. sp. (Digenea: Liolopidae) from intestine of serrated hinged terrapin, *Pelusios sinuatus* (Smith 1838) (Pleurodira: Pelomedusidae). (**3**) Female genitalia (holotype, USNM No. 1659278), ventral view. (**4**) Male genitalia (holotype, USNM No. 1659278), ventral view. Egg (e), ovary (o), oviduct (ov), ootype (oo), uterus (u), primary vitelline reservoir (pvr), dextral caecum (dc), transverse vitelline duct (tvd), sinistral caecum (sc), dextral excretory branch (deb), posterior vas efferens (pve), sinistral excretory branch (seb), posterior testis (pt), cirrus sac (cs), pars prostatica (pp), secondary bipartite internal seminal vesicle (sbisv), cirrus (c), initial bipartite internal seminal vesicle (ibisv), common genital pore (cgp), metraterm (m), vitellarium (vr), and vas deferens (vd).

**Figs. 5–6:**
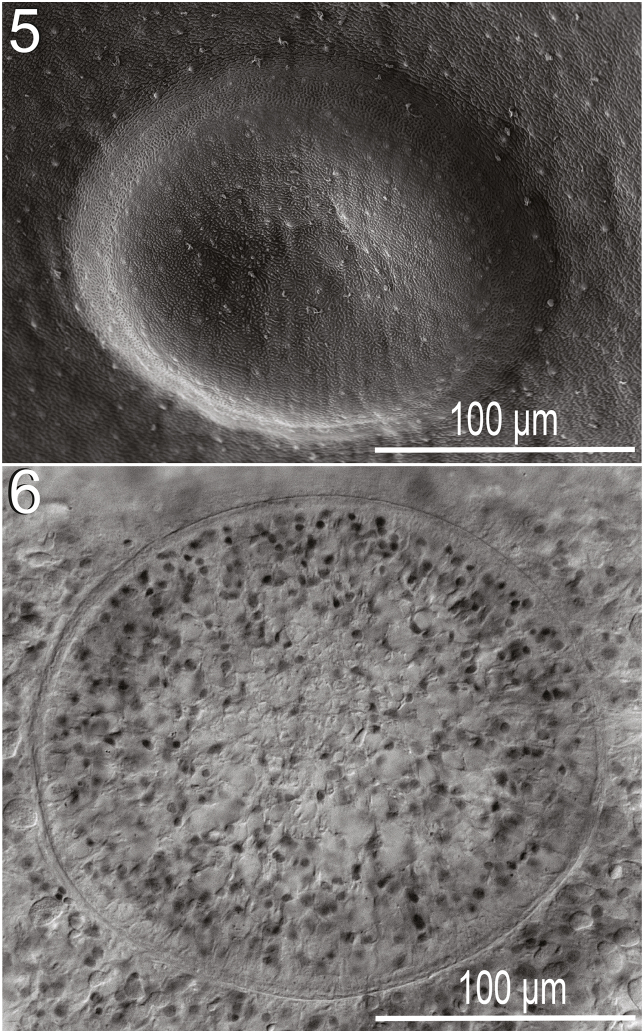
*Paraharmotrema karinganiense* Dutton & Bullard n. sp. (Digenea: Liolopidae) from intestine of serrated hinged terrapin, *Pelusios sinuatus* (Smith 1838) (Pleurodira: Pelomedusidae). (**5**) Ventral sucker, ventral view, scanning electron micrograph. (**6**) Ventral sucker, ventral view, light micrograph.

**Figs. 7–9:**
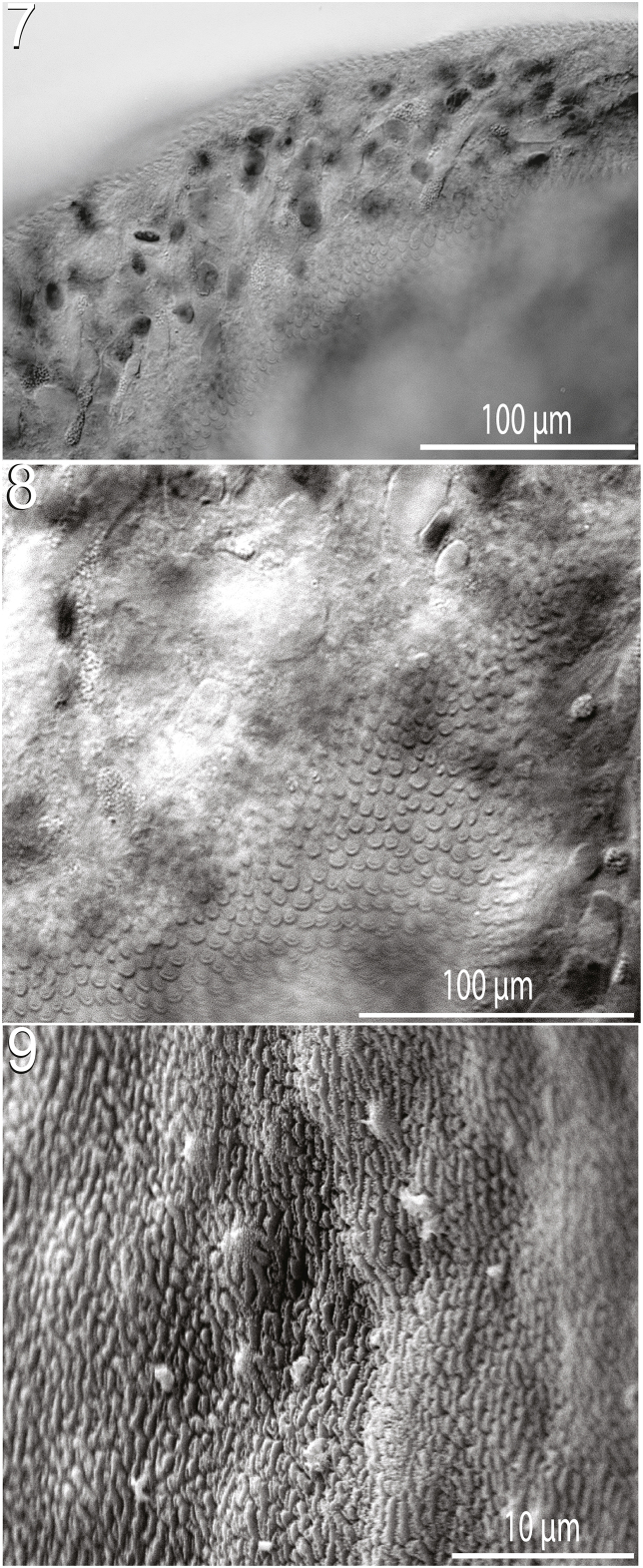
*Paraharmotrema karinganiense* Dutton & Bullard n. sp. (Digenea: Liolopidae) from the intestine of the intestine of the serrated hinged terrapin, *Pelusios sinuatus* (Smith 1838) (Pleurodira: Pelomedusidae). (**7**) Tegumental scales in antero-dextral ventral body surface, ventral view, light micrograph. (**8**) Tegumental scales on ventral body surface posterior to oral sucker, ventral view, light micrograph. (**9**) Tegumental scales in same position as in Fig. 8 (showing exposed tips of scales only), ventral view, scanning electron micrograph.

Anterior testis 340−640 (439 ± 97; 7) long or 4–5% (4% ± 1%; 6) of body length, 680−1000 (797 ± 113; 7) wide or 48–62% (53% ± 5%; 7) of body width at level of ovary; inter-testicular space 1500−2500 (1889 ± 372; 7) long or 17–22% (18% ± 2%; 6) of body length (Fig. 1). Posterior testis 365−1225 (554 ± 300; 7) long or 3–10% (5% ± 2%; 6) of body length, 640–840 (773 ± 68; 7) wide or 39–57% (52% ± 6%; 7) of body width at level of ovary, 1925−2950 (2368 ± 393; 7) or 22–24% (23% ± 1%; 6) of body length from posterior body end (Fig. 1). Anterior trunk of vasa efferentia emanating from ventral surface of anterior testis, extending anteriad 400−1150 (724 ± 267; 7), or 3–8% (6% ± 2%; 6), of body length, 13–25 (21 ± 4; 7) wide; posterior trunk of vasa efferentia emanating from ventral surface of posterior testis, extending anteriad 2000–3780 (2730 ± 585; 7), or 22–30% (27% ± 3%; 6), of body length, 15–35 (21 ± 7; 7) wide, meeting anterior trunk posterior to genital pore between anterior testis and cirrus sac to form vas deferens; vas deferens extending anteriad 350−500 (400 ± 58; 7) of body length, 15–25 (20 ± 3; 7) wide before turning sinistrad to insert into the bipartite seminal vesicle (Fig. 4). Cirrus sac semilunar, 575–900 (671 ± 110; 7) long or 5–7% (6% ± 1%; 6) of body length, 185–300 (216 ± 39; 7) wide or 13–20% (15% ± 2%; 7) body width at level of genital pore; cirrus 188–250 (220 ± 25; 7) long or 25–43% (33% ± 6%; 7) of cirrus sac length, 25–50 (41 ± 10; 7) wide; cirrus spines curved, tapering, 13–25 (20 ± 5; 7) long; cirrus associated with elongate gland cells and numerous prostatic cells (Fig. 4). Bipartite internal seminal vesicle enclosed within cirrus. Initial bipartite internal seminal vesicle oval, 125–275 (195 ± 52; 7) long or 1–2% (2% ± 0%; 6) of body length, 95–200 (140 ± 43; 7) wide, 1–2 × (1 ± 0; 7) longer than wide; elongate distal secondary bipartite internal seminal vesicle 205–520 (369 ± 100; 7) long or 2–4% (3% ± 1%; 6) of body length, 100–140 (109 ± 15; 7) wide, 2–4 × (3 ± 1; 7) longer than wide.

Ovary slightly lobed, 220–350 (266 ± 50; 7) long or 2–3% (2% ± 0%; 6) of body length, 225–325 (256 ± 32; 7) wide or 14–20% (17% ± 2%; 7) of body width; post-ovarian space 2175−4375 (3299 ± 708; 7) or 24–35% (31% ± 5%; 6) of body length (Fig. 3). Oviduct 125–230 (169 ± 37; 6) long or 1–2% (2% ± 1%; 5) of body length, 15–35 (27 ± 8; 6) wide, anterior to transverse vitelline duct, laterally expanding to form oötype. Laurer's canal not observed in wholemounts (using SEM, we did observe a pore on the ventral body surface between the level of the ovary and posterior testis that we suspect may be a Laurer's canal pore; a 2 μm wide pore accompanied by a tegumental depression having distinctive interconnected ridges). Vitellarium comprising a series of interconnected, irregularly-shaped masses of branched follicles wrapping around caeca and excretory system from posterior to ventral sucker to excretory vesicle, 88–125 (110 ± 15; 7) or 1% (1% ± 0%; 6) from posterior body end; transverse vitelline duct 750−1150 (941 ± 159; 6) in breadth, 15–50 (24 ± 13; 6) wide; primary vitelline collecting duct 325–500 (440 ± 58; 7) long, 100–155 (135 ± 20; 7) wide, inserting into oötype ventrally (Fig. 3). Oötype 100–175 (141 ± 26; 7) long, 15–35 (27 ± 8; 6) wide, between ovary and posterior testis (Fig. 3). Uterus convoluted, 2600−3970 (3099 ± 439; 7) in total length, 100–140 (114 ± 13; 7) wide, extending anteriad around sinistral margin of anterior testis. Eggs longer than wide, 130–160 (148 ± 9; 7) long, 85–100 (94 ± 6; 7) wide; number of eggs per specimen 28–55 (41 ± 12; 7). Metraterm 450–630 (539 ± 75; 7) long, 65–125 (84 ± 19; 7) wide; thick-walled, convoluted, surrounded by small glandular cells (Fig. 4). Uterus plus metraterm length 3100−4600 (3618 ± 508; 7) or 31–37% (34% ± 2%; 6) of body length. Common genital pore 4550−7900 (6307 ± 1123; 7) or 51–62% (59% ± 4%; 6) of body length from posterior body end, 35–85 (61 ± 16; 7) in diameter, ventral to sinistral caeca (Fig. 4).

Sinistral portion of excretory system 8450−11,135 (9563 ± 1165; 6) long, terminating 4100−7600 (5642 ± 1210; 6) or 46–65% (53% ± 8%; 6) of body length from anterior end; dextral portion of excretory system 8470−11,075 (9683 ± 1161; 6) long, terminating 3000–7435 (4805 ± 1639; 5) or 34–64% (46% ± 13%; 5) of body length from anterior end (Fig. 1−2).

#### 3.2.2 Taxonomic summary

*Type host: Pelusios sinuatus* (Smith, 1838) (Pleurodira: Pelomedusidae), serrated hinged terrapin; *other hosts: Pelusios subniger* (Bonnaterre, 1789) (Pleurodira: Pelomedusidae), east African black mud turtle; *Pelomedusa galeata* (Schoepff, 1792) (Pleurodira: Pelomedusidae), South African helmeted terrapin.

*Site in hosts:* Intestinal lumen.

*Type locality:* Karingani Game Reserve (KGR) (24°20′8.09″N 32°15′42.0″W), Maputo province, Mozambique; *other locality:* Roadside borrow pit filled with water (27°00′52.8″S 32°08′30.1″E), north-western Zululand, Kwa-ZuluNatal Province, South Africa (SA).

*Prevalence and intensity of infections:* 2 of 8 (25%) *P. subniger* from KGR were infected with 22 specimens of the new species; 1 of 3 (33%) *P. sinuatus* from KGR was infected with 1 specimen; 4 of 6 (66%) *P. galeata* from SA were infected with 13 specimens*.*

*Specimens and sequences deposited:* Holotype (USNM 1659278); paratypes (USNM 1659279−1659286); *nucleotide sequences*: *ex. P. galeata—* GenBank No. OL413006, OL413007 (ITS2); OL413003, OL413004 (28S); *ex. P. subniger—* GenBank No. OL413008 OL413008 (*ITS2*); OL413005 (*28S*); *Harmotrema laticaudae ex. L. semifasciata—* GenBank No. OL413009 (*28S*).

*Etymology*: The specific epithet “*karinganiense*” (neuter) is for the type locality and honors the personnel of KGR for their generous logistic support and cooperation in conducting parasite biodiversity research in Mozambique.

#### 3.2.3 Taxonomic remarks

The new genus can be easily differentiated from the other accepted genera of Liolopidae by the combination of having a linguliform body approximately 6–9 × longer than wide, tegumental spines/scales, a minute ventral sucker located in the anterior 1/7−1/8 of the body, deeply lobed testes that are transverse and abut the caeca (spanning the intercaecal space), a lobed ovary that is dextral and nearest the posterior testis, a uterus that is lateral to the anterior testis (not ventral to anterior testis), and a vitellarium that does not extend anteriad to the level of the ventral sucker and that does not fill the intercaecal space. The new species differs from *Helicotrema* spp. by having testes in the posterior half of the body; from *Liolope* spp. by having a cirrus sac that does not abut the ventral sucker; from *Harmotrema* spp. by having transverse, deeply lobed testes that abut the caeca; and from *Dracovermis* spp. by having testes that are far apart and that are not limited to the posterior 1/3 of the body (Table 2).

The new species is most similar to species of *Harmotrema* but further differs from all but one of them by having tegumental spines. The type species of *Harmotrema* (*H. infecundum*) and all congeners except *Harmotrema indica* Chattaparhyaya (1970) lack tegumental spines (Nicoll, 1914; Chattaparhyaya, 1970). Chattaparhyaya (1970) did not measure or draw a spine so we cannot know if the spines are similar/homologous to those of the new genus; we are skeptical that they are present in *H. indica*. The new species differs from *H. indica* and all congeners except *H. infecundum* by having a uterus that is lateral to the anterior testis; *H. infecundum* has a uterus that was illustrated as lateral to the anterior testis (Nicoll, 1914). The distribution of vitelline follicles further differentiates these taxa. In the new genus, the vitelline follicles do not extend anteriad beyond nor to the level of the ventral sucker, and the inter-caecal space posterior to the ventral sucker is predominantly void of vitelline follicles (Fig. 1). *Harmotrema infecundum* has vitelline follicles anterior to the ventral sucker (Nicoll, 1914); *H. laticaudae* has follicles that terminate at level of the ventral sucker and fill the inter-caecal space between the cirrus sac and ventral sucker (Yamaguti 1933); *H. eugari* has vitelline follicles that extend to nearly the level of the ventral sucker and fill the inter-caecal space (Tubangui and Masilungan, 1936); and *H. indica* has vitelline follicles that fill the intercaecal space between the ventral sucker and cirrus sac as well as between the posterior testis and excretory pore (Chattoparhyaya, 1970). The new genus further differs from *Harmotrema* spp. by having a large body >8 mm in length (vs. <7 mm), a minute ventral sucker not spanning the inter-caecal space (vs. ventral sucker spanning inter-caecal space), and a lobed ovary not abutting the posterior testis (vs. ovary not lobed, near to or abutting posterior testis). The new species also has a large uterus that can have >50 eggs (vs. 21 or less in *Harmotrema* spp.).

#### 3.2.4 Phylogenetic results

**Fig. 10:**
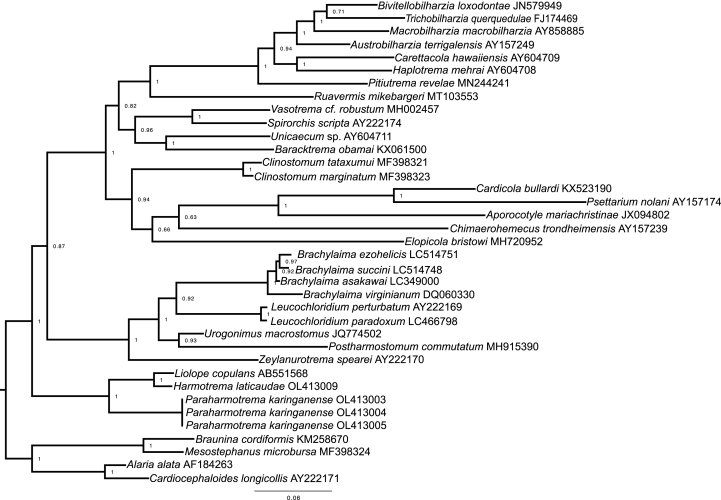
Large subunit ribosomal DNA (28S) Bayesian phylogeny. Values aside nodes are posterior probability. Scale bar is in substitutions per site. GenBank numbers in parentheses following each taxon.

All obtained *28S* and *ITS2* sequences from our specimens of *P. karinganiense* were identical, respectively. The *28S* sequences of the new species differed from *Liolope copulans* Cohn (1902) (AB551568) (Baba et al., 2011) by 104 (8%) nucleotides and from *H. laticaudae* by 105 (8%) nucleotides. The *28S* sequences of *H. laticaudae* and *L. copulans* differed by 38 (3%) nucleotides. Unsurprisingly based on morphology, the new species was recovered sister to the clade comprising *L. copulans* and *H. laticaudae* (Fig. 10). Our phylogenetic analysis recovered these liolopids sister to all other ingroup taxa analyzed and recovered the paraphyletic Brachylaimidae Joyeuz and Foley, 1930 with Leucochloridiidae Poche, 1907 as a clade sister to fish blood flukes (Aporocotylidae Odhner, 1912), clinostomes (Clinostomatidae Dollfus, 1931), turtle blood flukes (Spirorchiidae *sensu lato*), and schistosomes (Schistosomatidae Stiles and Hassall, 1898) (Fig. 10). Baba et al. (2011) recovered the 28S tree with the same topology as the present study, while Hernández-Mena et al. (2017) recovered *L. copulans* sister to the blood fluke clade, with the brachylaimid + leucochloridiid clade sister to the *L. copulans* + blood fluke clade in the *28S* tree. No *ITS2* sequence for another liolopid is public.

## 4 Discussion

The present study is the first record of a liolopid from South Africa or Mozambique, documents a second liolopid genus from *P. subniger*, which is the type and only known host for *Liolope dollfusi* Skrjabin (1962), and triples the number of liolopid turtle hosts reported from the continent of Africa (Table 1). The previous records of liolopid infections in turtles comprise a single species of *Liolope* (*L. dollfusi*) from a west African side-necked turtle (Pleurodira) plus two species of *Helicotrema* (*H. magniovatum* Odhner, 1912; *H. spirale* (Diesing, 1850) Odhner, 1912) from three neotropical turtles (two hidden-necked turtles (Cryptodira) and one side-necked turtle (Pleurodira)) (Table 1).

A robust phylogenetic hypothesis for Liolopidae has not been tested. The only study focused on the phylogenetic relationships among liolopid genera is the cladistic analysis of Brooks and Overstreet (1978) (hereafter, BO). The phylogenetic analyses of Baba et al. (2011), Hernández-Mena et al. (2017), and the present study comprise the only nucleotide-based phylogenies that include a liolopid. None has tested monophyly of liolopid genera nor robustly examined evolutionary relationships among the various liolopid lineages. The nucleotide-based studies lack the taxon sampling to assess phylogenetic interrelationships, with only a single taxon and two additional taxa included in the present study.

In their cladistic analysis based on 5 characters and the 4 accepted liolopid genera at that time, BO recovered *Liolope* sister to the other genera and *Dracovermis* sister to the crown group comprising *Harmotrema* and *Helicotrema*. They used this result to test hypotheses concerning host-parasite cophyly and biogeography. The cophyly analysis and its conclusions therein are problematic because (1) no tetrapod phylogeny is cited therein, (2) no cladistic matrix was provided (the character states assigned to the genera of Liolopidae can be inferred from the labelled cladogram but no matrix was published), (3) some character state assignments were erroneous, (4) exclusion of the turtle-infecting liolopid *L. dollfusi* was weakly justified (but excluding it was critical to supporting their hypothesis of cophyly), and (5) the other host records that would provide evidence to reject their cophyly hypothesis (i.e., the well-documented turtle-infecting liolopids) were wholly ignored or overlooked (see below). At least one example of an error cascade stemming from this work is evidenced by Niewiadomska (2002), who stated that no species of *Liolope* infects a turtle—perhaps relying upon BO to understand the diversity of hosts infected by liolopids.

Regarding liolopid-saurospidan cophyly, BO reported that, “*The parasites’ cladistic (genealogical) relationships reflect the phylogenetic relationships of their hosts*.” This was likely based on an antiquated understanding of the phylogenetic position of crocodylians (and *Dracovermis* was the focus of their paper) as closely related to lizards and snakes (Squamata), not as the sister lineage to birds (Aves) and member of the saurospidan crown group. Our current phylogenetic understanding of the natural history of reptiles and birds (Saurospida) (Chiari et al., 2012; Finn et al., 2014) is that frogs and salamanders (Amphibia) are the earliest branching lineage sister to the remaining saurospidans; with the lizards and snakes (Squamata) sister to the clade that comprises turtles (Testudines) sister to the crown group comprising crocodylians (Crocodylia) and birds (Aves). Mapping known liolopid infections onto that host phylogeny, *Liolope* spp. infect amphibians and turtles (which are not closely related); *Harmotrema* spp. infect water snakes, cobras, and sea kraits; *Helicotrema* spp. infect iguanas and turtles (each are members of early branching lineages that are distantly related to the crown group comprising crocodilians and birds); and *Dracovermis* infects crocodylians. The present study contributes a monotypic genus, *Paraharmotrema*, whose only species infects turtles.

The cladogram of BO clearly shows that the theorized phylogenetic relationships among liolopid genera therein does not allow for the acceptance of parasite-host cophyly under our modern understanding of vertebrate evolution. Most obvious is that crocodylians are apomorphic and sister to birds; they are not squamates (as presumed by BO). Hence, *Dracovermis* spp. (which infect crocodylians) must be recovered as apomorphic among liolopids if one is to accept parasite-host cophyly (no liolopid has been reported from a bird). No specimen of *Dracovermis* has been sequenced to date, but the phylogenetic position of this lineage is obviously intriguing. Cophyly would predict that *Dracovermis* spp. should be recovered as late branching, belonging to the crown group. Also obvious is that *L. dollfusi*, clearly diagnosed as a member of Liolopidae (see Niewiadomska (2002); inter-testicular ovary, ‘strigeid-like’ excretory system), infects a turtle but *Liolope* was recovered as stem lineage to the remaining liolopids in BO; clearly violating host-parasite cophyly (even from a coarse branching order perspective). In fact, these results alone reject monophyly of the turtle-infecting liolopids altogether. Interestingly, BO removed *L. dollfusi* from their analysis, regarding it as *incertae sedis* because they questioned the morphology of the oesophagus. However, this fluke is clearly a liolopid and infects a turtle. Hence, it is a lapse or intentional omission to exclude this host record in the context of host-parasite cophyly. BO made no argument for another genus assignment for this taxon within Liolopidae nor did they address this infection record in their discussion of host-parasite cophyly.

Regarding host records and the analysis of BO, as detailed above and including the new species, four liolopids (one, two, and one species of *Liolope*, *Helicotrema*, and *Paraharmotrema*, respectively; Table 1) infect turtles (Chelonia; a lineage sister to the clade including crocodylians and birds). These infections in turtles clearly violate a strict definition of liolopid-saurospidan cophyly (all records aside from the present study were published before 1978). In addition to dismissing *L. dollfusi* (see above), BO ignored or were evidently unaware of the other two turtle-infecting liolopids of *Helicotrema* that were known at that time (*H. magniovatum*; *H. spirale*). The existence of these turtle-infecting *Helicotrema* spp. further contradicts cophyly. In fact, based on the host records alone, one can see that *Liolope* and *Helicotrema* include species that lack phylogenetic host specificity, with each genus including species that collectively infect multiple saurospidan classes. This objectively makes a liolopid-saurospidan cophyly study seemingly pointless until more species are described.

The assignment of character states in BO is problematic and, in part, erroneous. Most important, not all species of the genera have all of the generic features presumably coded in their matrix. Their analysis was based on the minimum number of characters to run a cladistic analysis (n taxa – 1: oesophagus (present or absent), body shape (body length <4 × body width or body length >4 × body width), anterior extent of vitellaria (preacetabular or postacetabular), tegumental spines (present or absent), and gonad position (all or some extending into middle third of body or all contained in posterior third of body)). Below, we treat the problems or lapses with their use of the oesophagus, vitellarium, and scales/spines.

Regarding the oesophagus, we regard all liolopids as having a duct, i.e., an oesophagus, that courses through the pharynx and connects the mouth and intestine. Published descriptions show that this feature can be variable within a liolopid genus and open to interpretation—thereby making it a dubious character (based on published information) for inclusion in a cladistic matrix. BO evidently coded *Liolope* as lacking an oesophagus (after dismissing the only liolopid species that clearly has an elongate oesophagus; see above). They coded the remaining liolopid genera (*Harmotrema*, *Helicotrema*, *Dracovermis*) as oesophagus present (thereby comprising a critical synapomorphy that grouped those genera sister to *Liolope*—the theorized ancestral lineage; which was a critical piece of evidence that supported their cophyly hypothesis). However, *H. eugari* has an intestinal bifurcation that stems from the posterior margin of the pharynx, indicating that an oesophagus extending posteriad from the pharynx is absent, despite all other members of the genus having such an extension. Additionally, the posterior extent of the oesophagus and the location of the pharynx relative to the intestinal bifurcation in *Helicotrema* spp. is indeterminate based on the published illustrations of those species (Diesing, 1850; Odhner, 1912; Travassos, 1922). Moreover, BO's illustration of the body of *Dracovermis occidentalis* Brooks and Overstreet (1978) shows a laterally expanded portion of the intestinal bifurcation immediately posterior to the pharynx, i.e., no tube extends demonstrably posteriad beyond the pharynx (which could have been coded as “oesophagus absent”). To the contrary, *Dracovermis brayi* Brooks and Overstreet (1978) clearly has a long tube extending posteriad from the pharynx and connecting to the intestinal bifurcation (“oesophagus present”). BO included both species in their concept of the genus; seemingly inconsistent with their treatment of *L. dollfusi*.

Regarding the extension of the vitellarium, this too is inconsistent within liolopid genera. For example, BO coded *Harmotrema* as vitellarium post-acetabular; however, we have shown that the distribution of the vitelline follicles differentiates *Harmotrema* spp. from each other, i.e., they have an assortment of character states related to vitelline distribution (see Remarks). Additionally, they coded *Dracovermis* as having vitelline follicles that do not extend anterior to the ventral sucker; however, clearly, *Dracovermis nicollii* Mehra (1936) has vitelline follicles that extend far anterior to the ventral sucker. Its congeners have vitelline follicles that terminate at level of the ventral sucker or slightly anterior to the ventral sucker (Mehra, 1936; Tubangui and Masilungan, 1936; Baylis, 1940; Brooks and Overstreet, 1978). It would appear that their character state assignments, with exception to *L. dollfusi* and *Liolope*, were based on consensus among the congeners rather than confirming that each species of a genus has the character state they assigned to each genus included in their analysis.

Regarding tegumental scales (spines), BO evidently assigned the wrong character states to some of the genera in their analysis. *Liolope* spp., *H. indica*, and *P. karinganiense* have scales/tegumental spines, whereas the other liolopids lack them. Perhaps a lapse, BO (Fig. 4 therein) depicted an “*evolutionary shift of character state*” (character no. 4, presence/absence of tegumental spines) for *Helicotrema*; indicating that only *Helicotrema* lacks scales/tegumental spines. This is a critical error in character state assignment because it artifactitiously polarized *Helicotrema* from the remaining ingroup taxa, most of which lack spines. It also erroneously related *Dracovermis* to an earlier branching ancestor. Collectively, these issues underscore the fact that a robust phylogenetic hypothesis for the genera of Liolopidae is lacking and needed because the one study that has been published has fundamental problems.

Given the diversity of potential hosts and the wide geographic distribution of known infections, Liolopidae is likely taxonomically under-sampled across salamanders, lizards, snakes, turtles, and crocodylians. Relatively little is known about liolopid infections especially in turtles, with only 3 publications total that have detailed an infection in a turtle (Odhner, 1912; Travassos, 1922; Skrjabin, 1962); only *L. dollfusi* and the new species reportedly mature in turtles exclusively (Table 1). This is astonishingly low sampling effort given the extremely high diversity of extant freshwater turtle species (Rhodin et al., 2017; Bullard et al., 2019).
